# Pediatric Sedation Assessment and Management System (PSAMS) for Pediatric Sedation in China: Development and Implementation Report

**DOI:** 10.2196/53427

**Published:** 2024-08-07

**Authors:** Ziyu Zhu, Lan Liu, Min Du, Mao Ye, Ximing Xu, Ying Xu

**Affiliations:** 1 Big Data Center for Children’s Medical Care, Children’s Hospital of Chongqing Medical University, National Clinical Research Center for Child Health and Disorders, Ministry of Education Key Laboratory of Child Development and Disorders, Chongqing, China; 2 Department of Anesthesiology, Children’s Hospital of Chongqing Medical University, National Clinical Research Center for Child Health and Disorders, Ministry of Education Key Laboratory of Child Development and Disorders, 20 Jinyu Avenue, Liangjiang New Area, Chongqing, 400014, China, 86 13983409393

**Keywords:** electronic data capture, information systems, pediatric sedation, sedation management, workflow optimization

## Abstract

**Background:**

Recently, the growing demand for pediatric sedation services outside the operating room has imposed a heavy burden on pediatric centers in China. There is an urgent need to develop a novel system for improved sedation services.

**Objective:**

This study aimed to develop and implement a computerized system, the Pediatric Sedation Assessment and Management System (PSAMS), to streamline pediatric sedation services at a major children’s hospital in Southwest China.

**Methods:**

PSAMS was designed to reflect the actual workflow of pediatric sedation. It consists of 3 main components: server-hosted software; client applications on tablets and computers; and specialized devices like gun-type scanners, desktop label printers, and pulse oximeters. With the participation of a multidisciplinary team, PSAMS was developed and refined during its application in the sedation process. This study analyzed data from the first 2 years after the system’s deployment.

**Implementation (Results):**

From January 2020 to December 2021, a total of 127,325 sedations were performed on 85,281 patients using the PSAMS database. Besides basic variables imported from Hospital Information Systems (HIS), the PSAMS database currently contains 33 additional variables that capture comprehensive information from presedation assessment to postprocedural recovery. The recorded data from PSAMS indicates a one-time sedation success rate of 97.1% (50,752/52,282) in 2020 and 97.5% (73,184/75,043) in 2021. The observed adverse events rate was 3.5% (95% CI 3.4%‐3.7%) in 2020 and 2.8% (95% CI 2.7%-2.9%) in 2021.

**Conclusions:**

PSAMS streamlined the entire sedation workflow, reduced the burden of data collection, and laid a foundation for future cooperation of multiple pediatric health care centers.

## Introduction

### Context

Procedural sedation in infants and children is in great demand for anxiety, pain, and motor control. Over the past decades, pediatric sedation has evolved into a multispecialty practice, with guidelines established by the American Society of Anesthesiologists (ASA), the American Academy of Pediatrics, and the International Committee for the Advancement of Procedural Sedation . However, there is currently no universally accepted optimal strategy for pediatric procedural sedation. Factors such as medical resources, patient volume, and health insurance systems may contribute to variations in criteria for administering sedation, methods for monitoring depth of sedation, qualifications of professionals performing sedation, and the choice of sedative agents [1-3]. The Children’s Hospital of Chongqing Medical University (CHCMU) is the largest pediatric center in Southwest China. The CHCMU had 1400 inpatient beds in 2019, which increased to 2480 in 2021, with more than 3 million outpatient visits annually [4]. The huge patient volume at CHCMU highlights the need for an electronic data capture and management system (EDCMS) that seamlessly integrates multiple sedation steps. Such a system ensures reliability and consistency, reduces documentation time, and allows more time and resources to be devoted to sedation services [4,5]. In addition, the system can flag high-risk patients (eg, ASA III patients) and monitor their sedation process in real time, facilitating quicker detection of potential risks. Smaller hospitals may also find this system beneficial, as it can enhance work efficiency and enable proper data management for comprehensive assessment and tracking of all records.

After a 2-month pilot deployment, the feasibility of the current system was proven, and it has been fully implemented since January 2020.

### Problem Statement

The demand for procedural sedation has significantly risen due to increased awareness of its importance [6-8]. Nevertheless, the current anesthesia system employed inside the operating room is incompatible with pediatric sedation outside the operating room. Other challenges include insufficient data collection and nonuniform management, which were initially deemed unavoidable owing to the large number of patients and limited pediatric personnel and resources for pediatric care. Moreover, a low ratio of medical personnel to patient is a long-standing issue for procedural sedation in many limited-income countries in Africa and Asia [9] as well as in many European countries [10].

### Similar Interventions

Traditional tools, such as Research Electronic Data Capture (REDCap) [11], were developed for data collection and lacked the features necessary to manage sedation services. Similarly, anesthesia systems designed for the surgical settings do not align with the requirements of outpatient sedation services. The newly implemented Pediatric Sedation Assessment and Management System (PSAMS) was designed to meet the unique needs of pediatric sedation services, optimizing both data management and clinical workflow.

## Methods

### Aims and Objectives

In this study, we aimed to develop a system that could meet the needs of pediatric sedation services and optimize both data management and clinical workflow. First, we introduced the development and implementation of the PSAMS. Second, we analyzed the one-time sedation success rate and the incidence of adverse events based on the data collected by the PSAMS.

### Blueprint Summary

**Figure 1.**
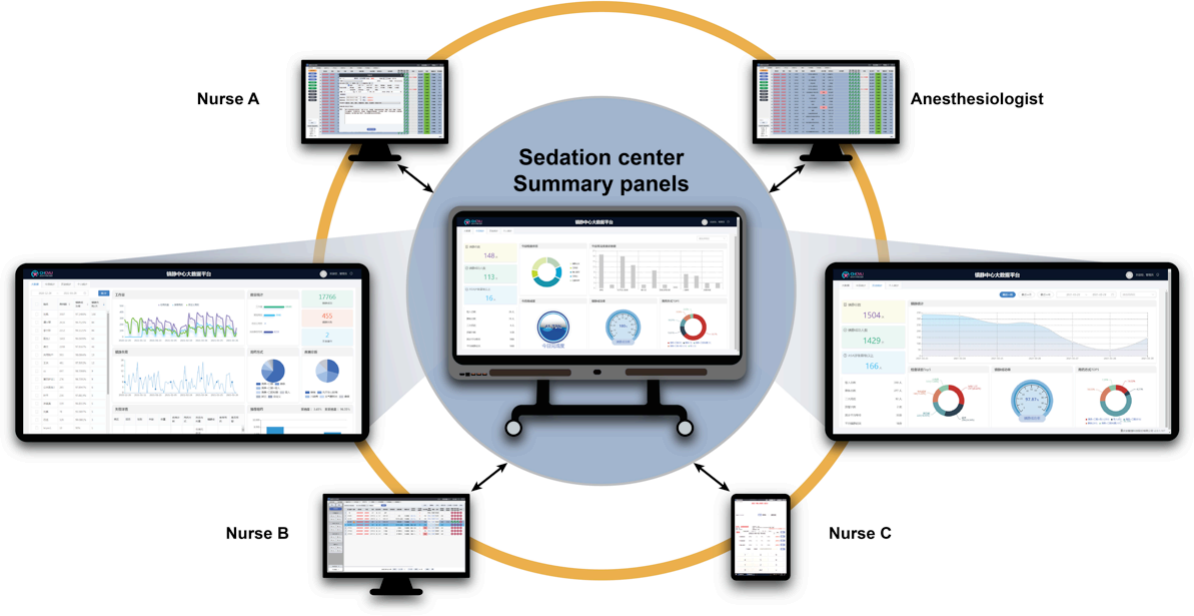
Devices and user interfaces of the Pediatric Sedation Assessment and Management System (PSAMS). All tasks are assigned to 4 roles by PSAMS: nurse A, anesthesiologist, nurse B, and nurse C. PSAMS is distributed on computers and portable tablets, and every user interface is specifically designed based on the different tasks of each user role. A large display in the sedation center provides a real-time update summary and statistics generated by PSAMS.

PSAMS was developed at the CHCMU based on the following principles: (1) user-friendly interfaces and features for anesthesiologists and nurses; (2) high accuracy with carefully designed error-proofing techniques; (3) interoperability and integration with the Hospital Information System (HIS); (4) compatible scalability reserved for future updates and to accommodate different deployments; and (5) solid security assurance on patients’ privacy. This system comprises 3 main parts: the software hosted on a server; clients distributed on portable tablets and computers; and devices, including gun-type scanners, desktop label printers, and pulse oximeters (Figure 1). This study adhered to the iCHECK-DH (Guidelines and Checklist for the Reporting on Digital Health Implementations) guidelines (Checklist 1) [12].

### Technical Design

The in-depth technical details of the construction of the PSAMS are available from the correspondence author upon reasonable request. Briefly, most of the system was written in C++; the Qt framework was used for application and graphical user interface development, and Java was used for back-end data processing. MySQL was applied as the database management system.

### Target

PSAMS has demonstrated great feasibility since its full implementation in 4 sedation centers of CHCMU in 2 districts of Chongqing from January 2020. PSAMS can be easily customized and adapted to other health care systems to provide sedation services for all pediatric patients (≤18 years of age). As of December 2022, it has been adopted by other health care centers in China, including Shenzhen Children’s Hospital in Guangdong Province and Zhengzhou Children’s Hospital in Henan Province, following team training and pilot implementation.

### Ethical Considerations

This study was approved by the Institutional Review Board of the Children’s Hospital of Chongqing Medical University (file number 2022, 220), and all data analyzed in this study were collected in accordance with its regulations. Informed consent was waived due to the retrospective observational nature of the study. Data will be stored on the hospital’s internal servers and managed by the hospital.

### Data

A comprehensive approach was implemented to ensure data quality. For data validation and verification, we designed and used prestructured data entry as drop-down menus or checkboxes. Other mandatory basic information fields were automatically synchronized with the HIS. Each data entry was immediately validated against predefined ranges or criteria. If an anomalous entry was detected (eg, 32 °C for body temperature or 400 kg for body weight), PSAMS would send notifications and remind the user to confirm the entry while recording the incident in the system’s running log, which also served as a dataset for analyzing error patterns and system performance issues over time. To further improve data integrity and support clinical decision making, PSAMS included advanced tools for automated dose calculation and sedation regimen recommendations, while aligning with best clinical practices. For example, the regimen recommendation tool was developed based on sedation practice and expert guidelines, allowing the anesthesiologist to accept or modify recommendations based on real-world scenarios. The regimen recommendation tool incorporated expert knowledge through a set of predefined rules considering factors such as age, weight, diagnosis, and procedure type. All tools were regularly updated and maintained based on user feedback and updated guidelines (Figure S1 in Multimedia Appendix 1). For adverse events reporting, the adverse event reporting tool from the World SIVA International Sedation Task Force [13] was integrated into PSAMS to facilitate early detection and provide standardized records.

### Interoperability

The PSAMS is integrated with the HIS, which applies the *International Classification of Diseases, Tenth Revision*. This interoperability ensures seamless data exchange and standardized coding for diagnoses, enhancing the accuracy and efficiency of medical records management within the system.

### Participating Entities

The multidisciplinary team included anesthesiologists and nurses from the Department of Anesthesiology, staff from the Department of Health Information Management, and software engineers. The Department of Health Information Management provided server, network, and HIS integration support. The software engineers created a prototype and refined the system based on feedback from the anesthesiologists and nurses during clinical practice. The funding body did not participate in the study design, implementation, or data governance.

### Budget Planning

Approximately 54% of the budget was spent on software development, 10% on change management, 25% on project management, 5% on user training, and 6% on product deployment. The development phase took 1 year in addition to a 2-month pilot deployment.

### Sustainability

PSAMS has been integrated into the HIS and used in several pediatric centers for procedural sedation. Therefore, PSAMS was maintained through the hospital budget and the remaining funds were dedicated to the active development and improvement of PSAMS.

## Implementation (Results)

### Implementation of PSAMS

**Figure 2.**
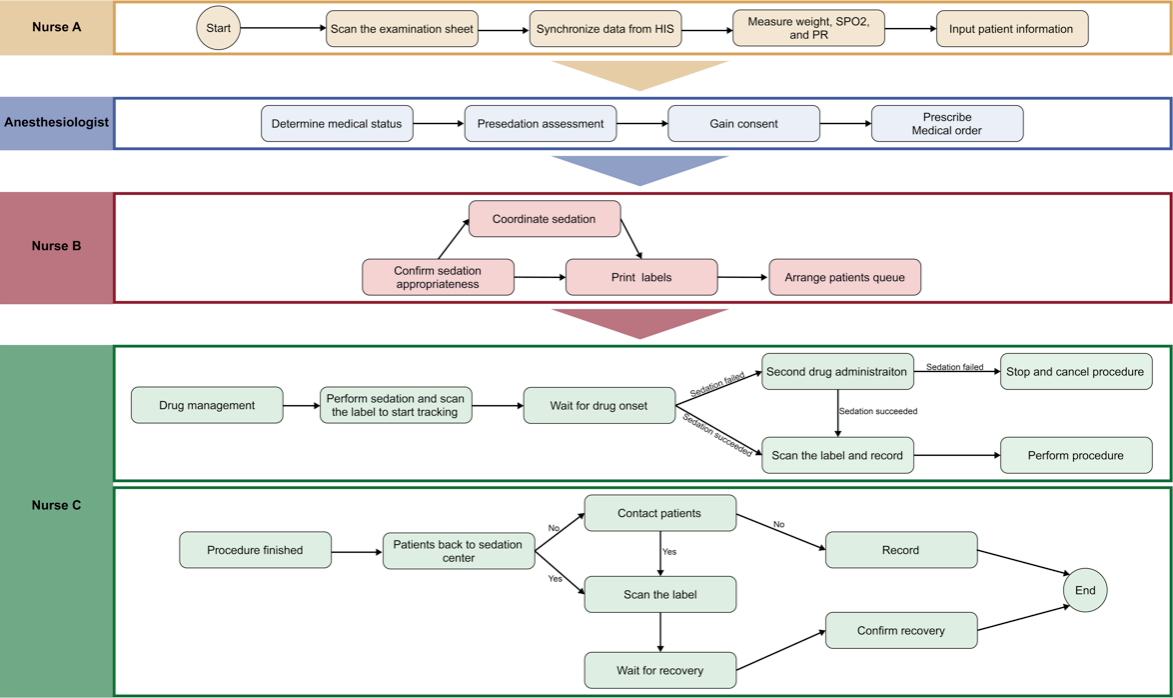
Complete sedation workflow mediated by the Pediatric Sedation Assessment and Management System in Children’s Hospital of Chongqing Medical University. HIS: Hospital Information System; PR:pulse rate; SpO_2_: saturation of peripheral oxygen.

Figure 2 shows the PSAMS-mediated sedation workflow. Patients arriving at the nurses’ station were registered by nurse A using a gun-type barcode scanner. This allowed automatic registration and synchronization of basic patient information (eg, name, age, gender, and ID) from the HIS. Previously, this process required manual entry of multiple forms, which typically took at least several minutes per patient. Following registration, nurse A quickly measured the patients’ body weight, temperature, pulse, and saturation of peripheral oxygen (SpO_2_), and completed the guidance sheet in PSAMS. The anesthesiologist then performed a presedation assessment based on physical examination and directed history (Table S1 in Multimedia Appendix 1). All relevant medical records were readily accessible via synchronization with the HIS, which was a significant improvement over the previous workflow, where data were isolated and difficult to retrieve. Patients deemed suitable for sedation who had signed informed consent were then directed to designated waiting or sedation areas.

From sedation to postprocedure recovery, pulse rate, and oxygen saturation were routinely recorded for ASA I and II patients at 4 time points: drug administration, sedation depth evaluation, procedure completion, and recovery from the procedure. Patients with ASA III and above received continuous monitoring of pulse rate and oxygen saturation by an experienced nurse with advanced life-support skills. PSAMS also tracked each step of the sedation process, displaying patient status on nurse dashboard panels, enabling nurse B to efficiently manage the patient queue and address any adverse events or complications promptly. In the case of an adverse event occurrence, PSAMS instantly alerted all screens within the sedation center, allowing the emergency team to quickly identify the potential reason and take appropriate measures to manage the adverse events. Meanwhile, the sedation records of the patients were immediately archived, synchronized, and reported to the department. Nurse C administered the drugs and logged the details into PSAMS. After the procedure, nurse C repeated the physical examinations and evaluated the recovery status. Overall, PSAMS had integrated previously independent sedation steps into a unified workflow, while maintaining and prioritizing important information, including vital parameters and sedation status at each stage.

### Overview of the Current Database of PSAMS

**Figure 3.**
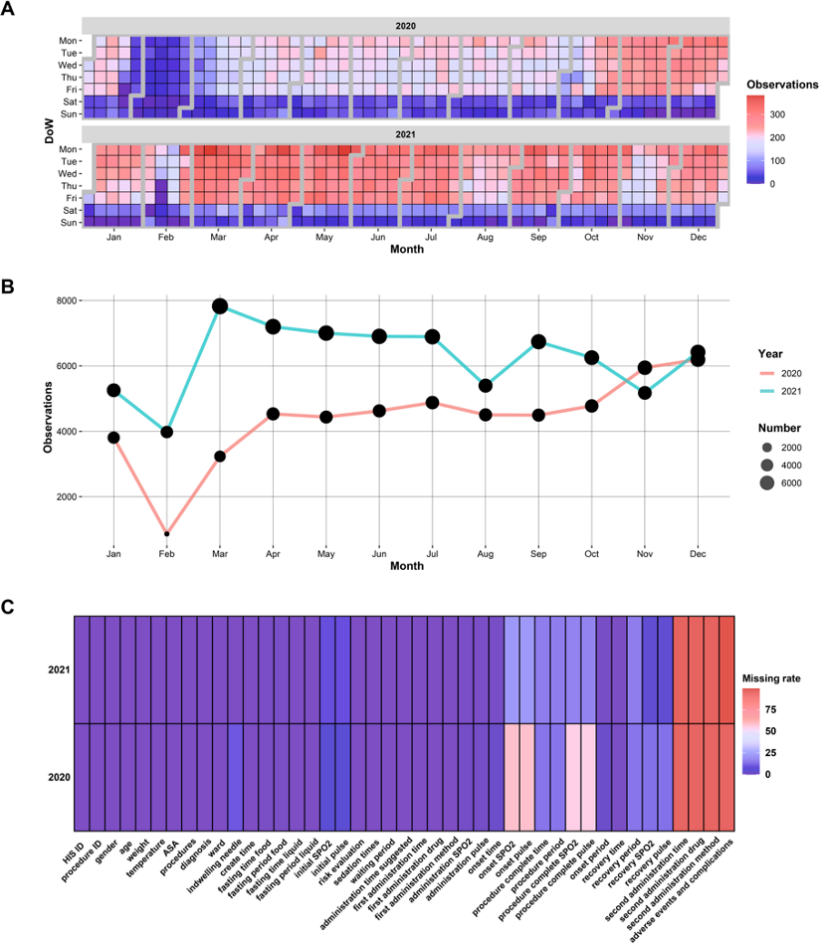
Overview of the sedation database from 2020 to 2021. (A) Calendar heatmap showing daily performed sedations from 2020 to 2021; (B) monthly summary of sedations from 2020 to 2021; (C) missing rate of each included variable in the database of the Pediatric Sedation Assessment and Management System. ASA: American Society of Anesthesiologists; SpO_2_: saturation of peripheral oxygen.

For a 2-year deployment from 2020 to 2021, a total of 127,325 sedations were performed in 85,281 patients (Figure 3A and B). In all, patients came from 31 provinces and municipalities in China (Table S2 in Multimedia Appendix 1). The year 2020 saw fewer sedations due to the COVID-19 pandemic, and February was notably affected by the Chinese lunar new year. We included 43 variables from presedation assessment (eg, body weight, temperature, and pulse) to postprocedural recovery (eg, postprocedural pulse, SpO_2_, and recovery time) for every single record (Table S3 in Multimedia Appendix 1). Of the 43 variables, 19 were complete in all records (Figure 3). High missing rates in some variables were due to the fact that most sedations did not require further intervention.

The demographic characteristics and ASA levels of the patients are summarized in Table 1. The mean age was 2.2 (SD 1.7) years, and the mean weight was 11.7 (SD 4.8) kg. The proportion of male patients (n=76,001, 59.7%) was higher than that of female patients (n=51,324, 40.3%). .

**Table 1.** Demographic characteristics and the American Society of Anesthesiologists (ASA) levels.

Demographic characteristics		Total (N=127,325)	Year 2020(n=52,282, 41.1%)	Year 2021(n=75,043, 58.9%)
**Age (year)**				
	Mean (SD)	2.2 (1.7)	2.1 (1.8)	2.3 (1.7)
	Range	0.0-18.0	0.0-17.9	0.0-18.0
**Gender, n (%)**				
	Female	51,324 (40.3)	21,325 (40.8)	29,999 (40.0)
	Male	76,001 (59.7)	30,957 (59.2)	45,044 (60.0)
**Weight (kg)**				
	Mean (SD)	11.7 (4.8)	11.5 (4.9)	11.9 (4.8)
	Range	1.0-61.0	1.5-61.0	1.0-60.0
**ASA, n (%)**				
	I	10,495 (8.2)	9134 (17.5)	1361 (1.8)
	II	108,136 (84.9)	39,196 (75.0)	68,940 (91.9)
	III	8568 (6.7)	3845 (7.4)	4723 (6.3)
	IV	125 (0.1)	106 (0.2)	19 (0.0)
	V	1 (0.0)	1 (0.0)	—^a^

a Not applicable.

### Sedative Choice, Procedures, Success Rate, and Adverse Events

PSAMS comprehensively collected and maintained information regarding the one-time sedation success rate and adverse events. The one-time sedation success was defined as achieving appropriate sedation depth with a single drug administration without adverse events. The most commonly used sedation regimen was dexmedetomidine combined with chloral hydrate (n=85,337, 67%) with a one-time sedation success rate of 96.6% (82,427/85,337; Table S4 in Multimedia Appendix 1). The most common procedure performed after sedation was magnetic resonance imaging (MRI; n=27,416, 21.5%), with the highest one-time sedation success rate of 99.7% (27,327/27,416; Figure S2 in Multimedia Appendix 1). The overall one-time sedation success rate was 97.1% (50,752/52,282) in 2020, which increased to 97.5% (73,184/75,043) in 2021. However, we were unable to summarize the one-time sedation success rate in 2019 and earlier because of the large volume of disorganized documents generated from the paper-based workflow and limited availability of personnel. Adverse events were recorded and reported using the adverse event reporting tool from the World SIVA International Sedation Task Force [13]. Most minimal risk adverse events, such as vomiting and hypersalivation, were not tracked and recorded by the PSAMS due to incompatibility with the monitoring devices. Minor risk adverse events were reported in 3397 sedations, where only 2 major adverse events were reported (Table 2).

**Table 2.** Adverse events reported in the Pediatric Sedation Assessment and Management System from 2020 to 2021.

Adverse events		Total (N=3962), n (%; 95% CI)	2020 (n=1843), n (%; 95% CI)	2021 (n=2119), n (%; 95% CI)
**Minimal adverse events**				
	Vomiting/retching	2 (0.1; 0-0.1)	1 (0; 0-0.2)	1 (0.1; 0-0.1)
	Subclinical respiratory depression	—^a^	—	—
	Hypersalivation	2 (0.1; 0-0.1)	2 (0.1; 0-0.3)	—
	Paradoxical response	—	—	—
	Recovery agitation	8 (0.2; 0.1-0.3)	8 (0.4; 0.1-0.7)	—
	Prolonged recovery	551 (13.9; 12.8-15.0)	295 (16; 14.3-17.7)	256 (12.1; 10.7-13.5)
**Minor adverse events**				
	Oxygen desaturation (75%‐90%) for <60 s	4 (0.1; 0-0.2)	2 (0.1; 0-0.3)	2 (0.1; 0-0.2)
	Apnea—not prolonged	—	—	—
	Airway obstruction	2 (0.1; 0-0.1)	2 (0.1; 0-0.3)	—
	Failed sedation	3389 (85.5; 84.4-86.6)	1530 (83; 81.3-84.7)	1859 (87.7; 86.3-89.1)
	Allergic reaction without asphyxia	—	—	—
	Bradycardia	1 (0; 0-0.1)	1 (0.1; 0-0.2)	—
	Tachycardia	1 (0; 0-0.1)	1 (0.1; 0-0.2)	—
	Hypotension	—	—	—
	Hypertension	—	—	—
	Seizure	—	—	—
**Major adverse events**				
	Oxygen desaturation—severe (<75% at any time) or prolonged (<90% for >60 s)	2 (0.1; 0-0.1)	1 (0.1; 0-0.2)	1 (0.1; 0-0.1)
	Apnea—prolonged (>60 s)	—	—	—
	Cardiovascular collapse/shock	—	—	—
	Cardiac arrest/absent pulse	—	—	—

a Not applicable.

## Discussion

### Principal Results

We developed the PSAMS through a multidisciplinary collaboration. To the best of our knowledge, This is the first EDCMS, specifically designed and used for pediatric sedation in China. PSAMS is deeply integrated with HIS. Thus, anesthesiologists could systematically determine a patient’s medical status and optimize the sedation techniques. In case of an adverse outcome or complication, PSAMS immediately called for backup assistance and reported the event. All the generated data were recorded and tracked by PSAMS. When transitioning to PSAMS, the biggest challenge for users was adapting to the new system, which was resolved after a 2-week training program. During this time, users encountered specific problems, such as unfamiliarity with the interface navigation, difficulty entering and saving data, or not knowing how to use the built-in tools. To address these challenges, we provided targeted support in the form of quick reference guides and on-demand lectures to facilitate a smoother transition. Meanwhile, PSAMS continues to be updated to improve usability and better meet the needs of users based on their feedback. Due to the previous poorly documented paper-based system, quantitatively comparison was unavailable, and a preliminary survey was conducted to evaluate the improvement after PSAMS implementation. Most users (19 out of 22 nurses and all 5 anesthesiologists) reported a reduction in administrative burdens, convenient access to patient data, and a smoother workflow, which allowed more focus on patient care per se. Overall, PSAMS has streamlined the entire workflow and aligned each member of the sedation team within a cohesive and structured process to ensure high-quality and efficient pediatric sedation.

### Limitations and Lessons Learned

PSAMS has several limitations. First, we did not perform real-time monitoring of every patient’s vital parameters, such as body temperature, respiration rate, pulse, and blood pressure, during the entire sedation process. In our clinical practice, current physiological monitoring systems failed to function while transferring patients and during examinations. To overcome this hurdle, new wearable monitoring devices, a highly networked information system, and a robust computing resource are necessary to achieve real-time monitoring. We are actively engaged in developing innovative wearable devices that integrate seamlessly with PSAMS and improve server performance with advanced algorithms and models. Second, many minimal adverse events were not recorded, and we believe that PSAMS can fill in this missing information after the implementation of new devices. Third, this study was performed at a single center; however, efforts are being made to extend PSAMS to other health care centers in China to prove its generalizability. Nevertheless, necessary adjustments and training are still required to ensure successful deployment of PSAMS in various settings. Additionally, PSAMS is vulnerable to mishandled data from the HIS, which means that the current system cannot yet verify the data imported from other databases.

In summary, valuable lessons have been learned from the PSAMS program. The key success factor is ensuring smooth communication within the multidisciplinary team, which keeps development on track and enables the realization of practical and user-friendly features. Additionally, allocating sufficient time for user training in advance is essential, as it accelerates the transition from the previous paper-based workflow. Finally, maintaining device redundancy is crucial, as devices such as pulse oximeters and tablets can be accidentally damaged during long-term deployment.

### Comparison With Prior Work

The advent of EDCMS and its integration with HIS has transitioned paper-based clinical workflow to an electronic one, significantly improved efficiency and data quality [14]. However, commonly used EDCMSs, such as REDCap, which provides hundreds of preconfigured forms to facilitate data capture, unfortunately, are not suitable for clinical sedation practice [15]. In our previous paper-based workflow, each step was separate, with no system for integrating and processing the information in one place. Nurses manually signed piles of medical paperwork, recorded patient status on notepads, and entered the information into a computer. Anesthesiologists performed sedation and spent more time preparing presedation assessment because of the cumbersome information exchange. The previous paper-based workflow had several limitations, such as the lack of a computerized system to integrate and process information in one place, cumbersome information exchange, and the inability to have a complete view of the entire sedation process. Consequently, nurses and anesthesiologists were compelled to allocate a significant proportion of their time on administrative tasks rather than patient care, and adverse events could be difficult to identify and were even ignored. Therefore, the application of well-computerized systems has been proposed as an effective way to reduce errors and improve the health care services [16,17].

Moreover, previous sedation databases only recorded data regarding sedation implementation, and the data were updated after a complete sedation workflow [18]. PSAMS included data from presedation assessment to postprocedural recovery and discharge and updated the database in a real-time manner. Consequently, the quickly accumulating data collected by PSAMS provided new insights into pediatric sedation.

At our institution, the most common procedure performed after sedation was MRI (n=27,416, 21.5%). Similarly, a retrospective analysis of 109,947 entries for MRI from the Pediatric Sedation Research Consortium, a large multicenter corroboration database containing more than 600,000 sedations, found that MRI was one of the most common imaging procedures requiring sedation [19]. Procedures like MRI and computed tomography (CT) scans require patients to remain still during imaging to ensure scanning quality, which can be difficult for children [20]. In fact, dexmedetomidine is used as a sole drug for noninvasive procedures, such as MRI and CT scans [21]. However, at our institution, we found that the dexmedetomidine alone cannot induce ideal sedation depth and often causes the children to awaken during or even before the MRI procedure. Therefore, a combination of rapidly titratable drugs and rapid onset drugs has been proposed as a standard regimen to improve sedation efficacy in China [4].

### Conclusions

In summary, we developed and applied PSAMS for pediatric sedation to meet the increasing demands. This is the first assessment and management system tailored for pediatric sedation. We are actively maintaining and improving PSAMS to optimize individualized sedation protocols, despite the heavy workload.

## Supplementary material

10.2196/53427 Multimedia Appendix 1 Additional Tables and Figures.

10.2196/53427 Checklist 1 iCHECK-DH (Guidelines and Checklist for the Reporting on Digital Health Implementations).

## Abbreviations

ASA: American Society of Anesthesiologists
CHCMU: Children’s Hospital of Chongqing Medical University
CT: computed tomography
EDCMS: electronic data capture and management system
HIS: Hospital Information System
iCHECK-DH: Guidelines and Checklist for the Reporting on Digital Health Implementations
MRI: magnetic resonance imaging
PSAMS: Pediatric Sedation Assessment and Management System
REDCap: Research Electronic Data Capture
SpO2: saturation of peripheral oxygen
