# Supplementary material for: Pediatric Sedation Assessment and Management System (PSAMS) for Pediatric Sedation in China: Development and Implementation Report
Source: JMIR Med Inform. 2024 Aug 7;12:e53427. doi: 10.2196/53427 (PMC11322794; doi:10.2196/53427)
Supplement: Multimedia Appendix 1 [file medinform-v12-e53427-s001.docx]

#
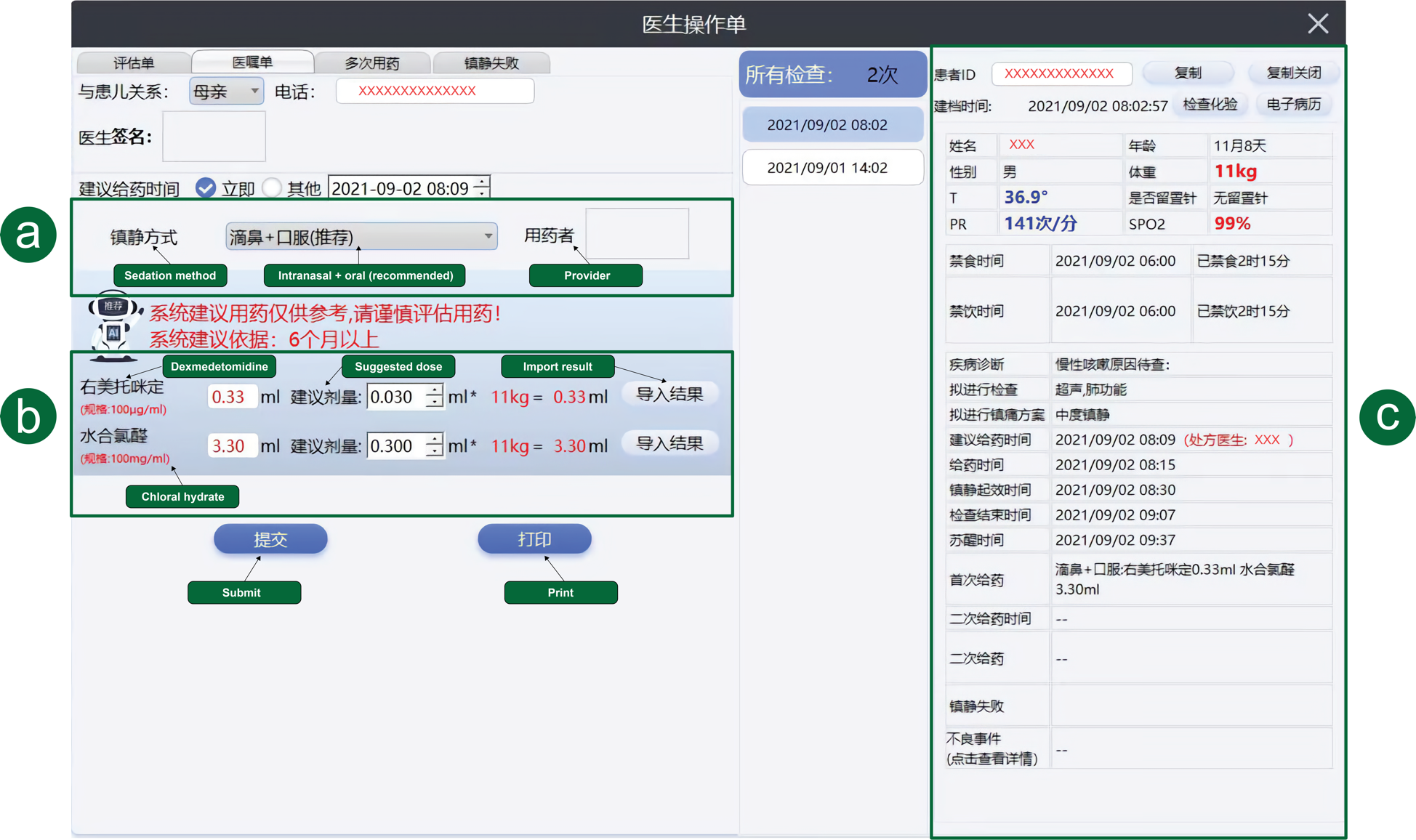
Figure S1. Screenshot of the anesthesiologist user interface in PSAMS

The user interface of the anesthesiologist is designed and implemented for maximal convenience and efficiency. (a) Dropdown menu of sedation methods; (b) built-in dose calculator for accurate dose calculation; (c) Review panel of patient’s information for comprehensive evaluation of underlying medical status.

#
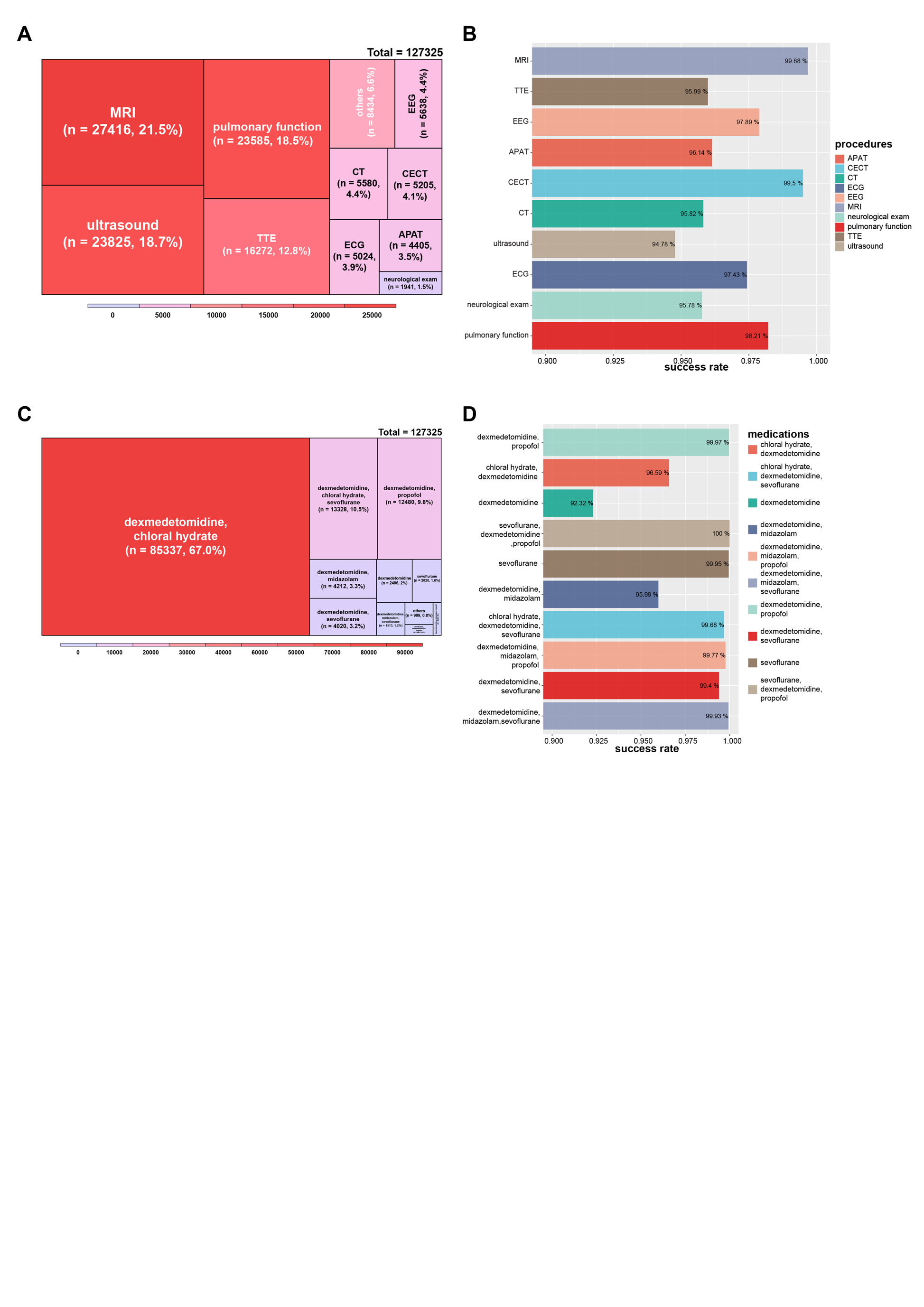
Figure S2. Sedation regimens and one-time sedation success rate

(A) Top ten most performed procedures after sedation and their proportions, respectively. (B) The one-time sedation success rates of the top ten most performed procedures. (C) Top ten most used sedation regimens and their proportions, respectively. (D) The one-time sedation success rates of the top ten most used sedation regimens.

**Table S1. Risk evaluation performed by the anesthesiologist in the evaluation part.**

|  | **Failure, N = 3,389^1^** | **Success, N = 123,936^1^** |
| --- | --- | --- |
| **Sedation history** |  |  |
| No | 1,746 (51.5%) | 74,855 (60.4%) |
| Yes | 1,643 (48.5%) | 49,081 (39.6%) |
| **Sedation failure history** |  |  |
| No | 3,066 (90.5%) | 120,957 (97.6%) |
| Yes | 323 (9.5%) | 2,979 (2.4%) |
| **Growth retardation** |  |  |
| No | 3,343 (98.6%) | 121,683 (98.2%) |
| Yes | 46 (1.4%) | 2,253 (1.8%) |
| **Congenital heart disease** |  |  |
| No | 2,926 (86.3%) | 110,752 (89.4%) |
| Yes | 463 (13.7%) | 13,184 (10.6%) |
| **Respiratory disease** |  |  |
| No | 2,938 (86.7%) | 105,832 (85.4%) |
| Yes | 451 (13.3%) | 18,104 (14.6%) |
| **Aspiration risk** |  |  |
| No | 3,376 (99.6%) | 123,582 (99.7%) |
| Yes | 13 (0.4%) | 354 (0.3%) |
| **Allergies** |  |  |
| No | 3,346 (98.7%) | 122,070 (98.5%) |
| Yes | 43 (1.3%) | 1,866 (1.5%) |
| **Deformities** |  |  |
| No | 3,108 (91.7%) | 115,202 (93.0%) |
| Yes | 281 (8.3%) | 8,734 (7.0%) |
| **Airway abnormalities** |  |  |
| No | 3,386 (99.9%) | 123,602 (99.7%) |
| Yes | 3 (0.1%) | 334 (0.3%) |
| **Cold limbs** |  |  |
| No | 3,389 (100.0%) | 123,934 (100%) |
| Yes | 0.0 (0.0%) | 2 (0.0%) |
| **Cyanosis** |  |  |
| No | 3,375 (99.6%) | 123,712 (99.8%) |
| Yes | 14 (0.4%) | 224 (0.2%) |
| **ASA** |  |  |
| 1 | 266 (7.8%) | 10,229 (8.3%) |
| 2 | 2,927 (86%) | 105,209 (84.9%) |
| 3 | 196 (5.8%) | 8,372 (6.8%) |
| 4 | 0 (0.0%) | 125 (0.1%) |
| 5 | 0 (0.0%) | 1 (0.0%) |
| ^1^n(%) |  |  |

**Table S2. Number of patients from 31 provinces and municipalities of China from 2020 to 2021.**

| **Provinces and municipalities** | **n** | **Percent (%)** |
| --- | --- | --- |
| Chongqing | 47393 | 55.57 |
| Sichuan | 23264 | 27.28 |
| Guizhou | 8330 | 9.77 |
| Yunnan | 1091 | 1.28 |
| Hubei | 496 | 0.58 |
| Hunan | 206 | 0.24 |
| Guangdong | 191 | 0.22 |
| Zhejiang | 151 | 0.18 |
| Henan | 138 | 0.16 |
| Jiangxi | 135 | 0.16 |
| Fujian | 128 | 0.15 |
| Gansu | 122 | 0.14 |
| Xinjiang | 109 | 0.13 |
| Xizang | 107 | 0.13 |
| Anhui | 94 | 0.11 |
| Shaanxi | 88 | 0.1 |
| Guangxi | 74 | 0.09 |
| Jiangsu | 63 | 0.07 |
| Shandong | 61 | 0.07 |
| Hebei | 59 | 0.07 |
| Beijing | 37 | 0.04 |
| Qinghai | 33 | 0.04 |
| Shanxi | 24 | 0.03 |
| Hainan | 23 | 0.03 |
| Liaoning | 22 | 0.03 |
| Inner Mongolia | 21 | 0.02 |
| Shanghai | 18 | 0.02 |
| Heilongjiang | 17 | 0.02 |
| Ningxia | 12 | 0.01 |
| Jilin | 10 | 0.01 |
| Tianjin | 5 | 0.01 |
| NA | 2759 | 3.24 |

**Table S3. An overview of the included variables in PSAMS.**

| **Variable name** | **Description** |
| --- | --- |
| HIS ID | The unique identifier of every patient in the HIS. |
| procedure ID | The unique identifier of every procedure |
| gender | Gender of current patient |
| age | Age of current patient (registered at the nurse station) |
| weight | Weight of current patient (registered at the nurse station) |
| temperature | Temperature of current patient (registered at the nurse station) |
| ASA | ASA level of the patient |
| procedures | Procedure name |
| diagnosis | Diagnosis of the patient |
| ward | Ward of the patient |
| indwelling needle | Whether the patient received indwelling needle |
| create time | Time when creating the record |
| fasting time food | Start time of fasting for food |
| fasting period food | Time length of fasting for food |
| fasting time liquid | Start time of fasting for liquid |
| fasting period liquid | Time length of fasting for liquid |
| initial SPO2 | SPO2 measured when patient arrived at the nurse station |
| initial pulse | Pulse measured when patient arrived at the nurse station |
| risk evaluation | Risk evaluation performed by anesthesiologist for sedation |
| sedation times | Total sedation times in PSAMS |
| waiting period | Time length of waiting for receiving sedation |
| administration time suggested | Suggested time to administrate drugs |
| first administration time | Time of the first-time administration of drugs |
| first administration drug | Name of the first-time administration of drugs |
| first administration method | Administration method of the first-time administration of drugs |
| administration SPO2 | SPO2 measured when patient receiving sedation |
| administration pulse | Pulse measured when patient receiving sedation |
| onset time | Time when drugs took effect |
| onset SPO2 | SPO2 measured when drugs took effect |
| onset pulse | Pulse measured when drugs took effect |
| procedure complete time | Time when procedure is finished |
| procedure period | Time length for performing the procedure |
| procedure complete SPO2 | SPO2 measured when the procedure is completed |
| procedure complete pulse | Pulse measured when the procedure is completed |
| onset period | Time length for drugs to take effect |
| recovery time | Time when patient starts recovering from sedation |
| recovery period | Time length for patient to recovery from sedation |
| recovery SPO2 | SPO2 measured when the patient starts recovering from sedation |
| recovery pulse | Pulse measured when the patient starts recovering from sedation |
| second administration time | Time of the second-time administration of drugs |
| second administration drug | Name of the first-time administration of drugs |
| second administration method | Administration method of the second-time administration of drugs |
| adverse events and complications | Adverse event and complications happened during sedation |

| **n (%)** | **anorectal exam,**  **N=423^1^** | **APAT,**  **N=4,405^1^** | **CECT,**  **N=5,205^1^** | **CT,**  **N=5,580^1^** | **ECG,**  **N=5,024^1^** | **EEG,**  **N=5,638^1^** | **IOP,**  **N=218^1^** | **MRI,**  **N=27,416^1^** | **neurological exam,**  **N=1,941^1^** | **others**  **N=7,793^1^** | **pulmonary function**  **N=23,585^1^** | **TTE**  **N=16,272^1^** | **ultrasound**  **N=23,825^1^** |
| --- | --- | --- | --- | --- | --- | --- | --- | --- | --- | --- | --- | --- | --- |
| chloral hydrate,  dexmedetomidine | 416 (98%) | 4,309 (98%) | 162 (3.1%) | 4,482 (80%) | 4,476 (89%) | 4,951 (88%) | 177 (81%) | 716 (2.6%) | 1,848 (95%) | 5,496 (71%) | 23,163 (98%) | 13,881 (85%) | 21,260 (89%) |
| chloral hydrate,  dexmedetomidine,  sevoflurane | 2 (0.5%) | 24 (0.5%) | 2,615 (50%) | 181 (3.2%) | 17 (0.3%) | 15 (0.3%) | 2 (0.9%) | 8,895 (32%) | 10 (0.5%) | 1,078 (14%) | 19 (<0.1%) | 116 (0.7%) | 354 (1.5%) |
| dexmedetomidine | 0 (0%) | 4 (<0.1%) | 1 (<0.1%) | 100 (1.8%) | 254 (5.1%) | 635 (11%) | 2 (0.9%) | 5 (<0.1%) | 41 (2.1%) | 42 (0.5%) | 18 (<0.1%) | 143 (0.9%) | 1,241 (5.2%) |
| dexmedetomidine,  midazolam | 5 (1.2%) | 57 (1.3%) | 156 (3.0%) | 316 (5.7%) | 233 (4.6%) | 10 (0.2%) | 4 (1.8%) | 716 (2.6%) | 24 (1.2%) | 452 (5.8%) | 366 (1.6%) | 1,507 (9.3%) | 366 (1.5%) |
| dexmedetomidine,  midazolam,  propofol | 0 (0%) | 0 (0%) | 60 (1.2%) | 3 (<0.1%) | 0 (0%) | 2 (<0.1%) | 0 (0%) | 332 (1.2%) | 0 (0%) | 31 (0.4%) | 0 (0%) | 0 (0%) | 7 (<0.1%) |
| dexmedetomidine,  midazolam,  sevoflurane | 0 (0%) | 0 (0%) | 110 (2.1%) | 11 (0.2%) | 0 (0%) | 0 (0%) | 0 (0%) | 1,234 (4.5%) | 0 (0%) | 79 (1.0%) | 0 (0%) | 39 (0.2%) | 40 (0.2%) |
| dexmedetomidine,  propofol | 0 (0%) | 0 (0%) | 1,742 (33%) | 59 (1.1%) | 0 (0%) | 1 (<0.1%) | 0 (0%) | 10,255 (37%) | 0 (0%) | 379 (4.9%) | 0 (0%) | 28 (0.2%) | 16 (<0.1%) |
| dexmedetomidine,  sevoflurane | 0 (0%) | 9 (0.2%) | 236 (4.5%) | 354 (6.3%) | 24 (0.5%) | 14 (0.2%) | 2 (0.9%) | 2,335 (8.5%) | 13 (0.7%) | 148 (1.9%) | 12 (<0.1%) | 427 (2.6%) | 446 (1.9%) |
| sevoflurane | 0 (0%) | 0 (0%) | 7 (0.1%) | 13 (0.2%) | 1 (<0.1%) | 0 (0%) | 1 (0.5%) | 1,975 (7.2%) | 0 (0%) | 8 (0.1%) | 0 (0%) | 3 (<0.1%) | 12 (<0.1%) |
| sevoflurane,  dexmedetomidine,  propofol | 0 (0%) | 0 (0%) | 2 (<0.1%) | 0 (0%) | 0 (0%) | 0 (0%) | 0 (0%) | 487 (1.8%) | 0 (0%) | 6 (<0.1%) | 0 (0%) | 0 (0%) | 0 (0%) |
| others | 0 (0%) | 2 (<0.1%) | 114 (2.2%) | 61 (1.1%) | 19 (0.4%) | 10 (0.2%) | 30 (14%) | 466 (1.7%) | 5 (0.3%) | 74 (0.9%) | 7 (<0.1%) | 128 (0.8%) | 83 (0.3%) |

**Table S4.** **The proportions of different sedation regimens in each procedure**
