# Supplementary material for: Pediatric Sedation Assessment and Management System (PSAMS) for Pediatric Sedation in China: Development and Implementation Report
Source: JMIR Med Inform. 2024 Aug 7;12:e53427. doi: 10.2196/53427 (PMC11322794; doi:10.2196/53427)
Supplement: Checklist 1 [file medinform-v12-e53427-s002.docx]

Checklist of iCHECK-DH guidelines. iCHECK-DH: Guidelines and Checklist for the Reporting on Digital Health Implementations.

| section |  | Item | Description |
| --- | --- | --- | --- |
| Title | 1 | Title  (M^[[1]](#footnote-1)^) | Pediatric Sedation Assessment and Management System (PSAMS) for Pediatric Sedation in China: Development and Implementation Report |
| Abstract | 2 | Abstract  (M) | See in Abstract. The PSAMS improved pediatric sedation services by streamlining workflow and reducing data collection burden at a major children's hospital in southwest China. |
| Introduction | 3 | Context  (M) | See in Introduction section. |
|  | 4 | Problem statement  (M) | See in Introduction section |
|  | 5 | Similar Interventions  (M) | See in Introduction section |
| Methods | 6 | Aims and Objectives  (M) | See in Methods section |
|  | 7 | Blueprint summary  (M) | See in Methods section |
|  | 8 | Technical Design  (M) | See in Methods section |
|  | 9 | Target  (M) | See in Methods section |
|  | 10 | Data  (M) | See in Methods section |
|  | 11 | Interoperability  (M) | See in Methods section |
|  | 12 | Participating entities  (M) | See in Methods section. A multidisciplinary team consisting of anesthesiologists, nurses, health information management staff, and software engineers collaborated to develop the PSAMS. |
|  | 13 | Budget Planning  (M) | See in Methods section |
|  | 14 | Sustainability  (M) | See in Methods section |
| RESULTS | 15 | Coverage  (M) | See in Results section (Implementation of the PSAMS). From January 2020 to December 2021 |
|  | 16 | Outcomes  (M) | See in Results section (Overview of the current database of PSAMS) and Discussion section (Principal Results) |
|  |  | Lessons learned  (M) | See in Discussion section (Limitations, and Lessons Learned) |
|  | 17 |  |  |
|  | 18 | Unintended consequences  (NM^[[2]](#footnote-2)^) | NA |
| Discussion | 19 | Conclusion  (M) | See in Conclusion section |
| General | 20 | General  (NM) | NA |

1. M: Mandatory item [↑](#footnote-ref-1)
2. NM : Non-mandatory item [↑](#footnote-ref-2)
